# Book Reviews

**Published:** 1814-06

**Authors:** 


### CRITICAL ANALYSIS

### OF RECENT PUBLICATIONS

IN THE

### DIFFERENT BRANCHES OF PHYSIC, SURGERY, AND MEDICAL PHILOSOPHY.

---

*A Practical Account of the Fever commonly called the Bilious Remittent, as it appeared in the Ships and Hospitals of the Mediterranean Fleet; with Cases and Dissections. To which are added, Facts and Observations, illustrative of the Causes, Symptoms, and Treatment of Fever, in the Mediterranean; comprehending the History of Fever in the Fleet during the Years* 1810, 1811, 1812, 1813; *and of the Gibraltar and Carthagena Fevers.* By WILLIAM BURNETT, M.D. Physician of the Fleet, late Physician and Inspector of Hospitals to His Majesty's Fleet in that Sea. 8vo. pp. 296. Callow, 1814.

THE existence of local inflammation as a frequent cause of fever, though established on the most undeniable evidence, has not received that attention from practitioners which the importance of the fact demands. Sensible of the value of blood-letting in many fevers, and particularly in those of warm climates, we are happy to find this doctrine so ably maintained in the volume before us, which contains abundant and conclusive evidence on the subject. We trust the time is now arrived when the brown furred tongue and the crusted tooth will no longer be considered the characteristics of putrescence, or the prostration of strength be deemed sufficient in itself to contra-indicate the use of the lancet.

It is curious to remark the great feeling of debility and tendency to fainting which existed in our author's cases under circumstances where bleeding was indispensable. The disease in most instances commenced with lipothymia, when the victims suddenly fell down with giddiness. The *pulse could scarcely be felt*, and yet these alarming symptoms immediately gave way to liberal and copious bleedings. The patient, who came in supported, or carried by men, after the abstraction of twenty ounces of blood, got up and walked, wondering at his relief. Venesection was the sovereign and speedy remedy in alleviating their sufferings, producing a perfect remission in twenty-four hours, the blood always exhibiting strong inflammatory marks. One man lost

198 ounces of blood before the fever was subdued, and this had so little effect in producing *debility*,* that the man was on duty in three weeks.

Advocates as we are for the practice of depletion, let it not be supposed that we consider this remedy applicable to *all* fevers. No man in his senses can maintain such a proposition who has read the admirable description of the nervous fever by Huxham; but it cannot be denied that many cases which have received the fashionable though insignificant name of typhus, are in reality the symptoms of local disease, whose seat varies in different patients. On this part of the subject we shall content ourselves with giving a general hint, leaving it to our brethren to discriminate between the cases in which bleeding is or is not necessary. The following is the author's description of the fever, with the dissections *post mortem.*

"Towards the end of June, or commencement of July, slight attacks of fever begin to present themselves; the patient complains of considerable head-ach, with nausea and prostration of strength; the eyes are somewhat suffused, and the countenance a little flushed; the tongue is white and moist, and he has considerable thirst; the skin is at times moist, and the temperature little increased; at others it is dry, and the heat pungent. The pulse is sometimes full and strong, beating at the rate of 120 in the minute; in others it is less so; and in some the increase of velocity is scarcely perceptible: there is commonly constipation of the bowels, and loss of appetite. Though this is, for the most part, the appearance of the fever of the summer on its first attack, yet in many instances it has been found to put on a far more serious aspect, and assume the form of the severer degree of this fever, which occurs in the autumn. This, however, has in general been traced to some irregularity committed by the patient, as exposure to the sun or night dews; or else to the plan of treatment adopted at the commencement of the disease. At this time the gastric symptoms are seldom severe, the head being the viscus most materially affected, and to the relief of this, and the free evacuation of the intestines, must the principal attention of the medical attendant be directed. As the summer advances, the attacks become more formidable, and accordingly require the most prompt and efficacious treatment.

"The patient first feels a degree of lassitude and prostration of strength, (in some the latter appears very considerable); this is succeeded by a sense of chilliness, extending along the spine and lumbar region, which is followed by increased heat and severe head-ach,

* It is of great importance to attend to the distinction between depression of strength and actual debility. The former has not unaptly been compared to a spring depressed by a weight, which returns to its former elasticity and power when the weight is removed.

referred referred chiefly by the patient to the forehead and temples; and in the severer cases it extends in the course of the longitudinal sinus. A deep-seated pain in the orbits is also experienced; the eyes are sometimes unnaturally prominent, with a watery inflammatory appearance, and impatience of light. There is often a very considerable degree of redness and tumefaction of the face, the skin having a glossy shining appearance; the flushing of the face frequently extending downward, as far as the superior part of the sternum: the tongue is white or slightly yellow, and commonly moist, with a bad taste in the mouth. There is a sense of uneasiness in the epigastric region, with nausea, and in some patients a vomiting of a bilious-like matter; pains in the joints, back, and calves of the legs, disturbed sleep, and constipation of the bowels, are amongst the symptoms usually observed. The pulse for the most part is full and hard, though not always, particularly where the gastric symptoms are severe; at other times it is oppressed, but rises under the lancet. In its first state, which generally accompanies the severe local affection of the brain, it beats from 110 to 120 in the minute, sometimes upwards of 130, but at this period it is not a very common occurrence. In its second stage, which generally accompanies a more advanced stage of the attack, the pulse seldom exceeds 90 or 100, and is not so full; nay, is frequently observed to be slower than natural; there is generally a throbbing of the carotid and temporal arteries, with great thirst and considerable anxiety. The superior parts of the body are sometimes covered with a profuse perspiration, but generally the skin is dry, and the temperature increased; if the disease be advanced, the heat is often pungent, and there is through its whole course a loathing of food. Severe rigors sometimes, but not very commonly, precede the hot stage of the disease.

"In many cases, however, this fever makes its attack without any very sensible previous indisposition, and in several instances, the patient, while at his usual work, has dropped down in a state of insensibility; but this stage of the disease seldom lasts long, re-action takes place with increased circulation, and the most marked symptoms of determination to the brain. During the winter months, the morbid affection of the brain is not at all times so prominent a symptom, nor are the bowels so much constipated: there is often a more anxious look in the beginning, some pain upon pressing the abdomen, and the pulse is seldom so full, when the last symptom is considerable. The attack has also been accompanied by cynanche tonsillaris, but this soon ceases to be a prominent symptom. In the summer and autumnal months, an attack of cholera morbus, or diarrhœa, has preceded this fever.

"If the patient has not complained till the second or third day of his illness, if the disease has been treated as a typhoid affection, or when the attack is violent, if the most prompt means have not been adopted, the patient will commonly have a different appearance. The head-ach is still severe, but accompanied by stupor, disinclination to answer questions, and indifference to surrounding objects: the eyes have a duller look than usual, and frequently the preceding

 inflammatory inflammatory appearance, has in some measure given way to a slight yellowness of the tunica adnata, which soon extends to the face and neck, and often, in twenty-four hours from this time, to the whole body. The tongue is now covered with a thick yellow coat, or is brown and dry in the middle, the edges having a red inflammatory appearance; the prostration of strength is considerable; the anxiety and pain in the limbs great; the uneasiness in the epigastric region is urgent; and there is frequent vomiting of a bilious-like matter, and most harassing singultus; the pulse under these circumstances is commonly much smaller, varying from 100 to 120, and often is more frequent. There is at this time no very considerable increase of temperature, the features exhibiting rather a shrunk and anxious appearance, with occasional partial flushings. In the severe attacks, about the third day, there is often an appearance of a complete remission, but the evening puts an end to the delusion; an exacerbation takes place, with great increase of all the dangerous symptoms. Unhappily this deceitful period has often been mistaken for a real remission of the symptoms, and both tonics and stimulants have been given, with a view to prevent a recurrence of the paroxysm; but vain, indeed, are all such efforts,—they serve but to increase the malady.

" As the disease advances, the pain and uneasiness about the epigastric region continue to increase. There is now almost constant vomiting, considerable pain upon pressure, great restlessness, with oppression at the præcordia. The abdomen is likewise painful, with frequently thin, black, fœtid, and sometimes gelatinous-like stools. The suffusion, at first of a bright yellow, now assumes a darker hue; the skin is at times moist, or there are partial sweats; and commonly a disagreeable fœtor is exhaled from the person or linen of the patient. Under all these distressing circumstances he often retains his intellect, answering questions rationally; but there is almost always some degree of wandering, and inattention to surrounding objects. If the head has been materially affected, there is occasionally considerable delirium, which commonly terminates in a state of coma. The pulse becomes irregular, sometimes full, at others quick and small, and often intermitting. The uneasiness about the epigastric region is intolerable, and some patients have complained of a burning sensation, extending upwards to the throat. The vomiting is incessant, often of blood, succeeded, *in some cases*, by a matter resembling coffee grounds; blood exudes from the gums and fauces, with hæmorrhage from the nose, and often in considerable quantities from the anus. About this time subsultus tendinum comes on, with picking of the bed-clothes, constant tossing in the bed, and suppression of urine, with an irksome pain across the pubes. In many cases complete ischuria renalis; in a few, the bladder has been found distended, and required the introduction of a catheter; in many, the stools are passed involuntarily. Swelling and suppuration of the parotids, petechiæ and vibices, though not general symptoms, have in several cases occurred. The tongue is now covered by a black crust, the teeth are surrounded by sordes, the breathing becomes more laborious, with with great action of the respiratory muscles. The anxiety is extreme; the pulse sinks so as to be sometimes scarcely perceptible, and intermits; cold extremities, clammy sweats terminate the scene, frequently on the third or fourth, but generally from the fifth to the eighth day; though sometimes death is protracted beyond that period.

"These are the symptoms which mark the progress of this fever, under its most formidable mode of attack, or when it has been neglected or improperly treated. In many instances it proceeds through its whole course, bearing strictly the form of a continued fever; in others there is a deceitful remission about the third day. But in by far the greater number of cases, though there are evening exacerbations, there is but seldom any evident and clear remission in the morning. The most attentive observation by myself, and others on whom I could rely, has failed to detect the distinct remissions ascribed to this disease by Dr. Cleghorn.

"The train of symptoms which have been just enumerated, will not always be presented in the same patient, nor is death itself uniformly preceded by such appearances as I have described, particularly when the disease terminates fatally before the third day, or before the first stage of the disease is past. In such cases the brain is the part more immediately affected, and death has sometimes taken place suddenly; the pulse remaining full, soft, and even somewhat strong, till nearly the last moments.

"I have before observed, that during the early part of summer, the brain is the organ most violently attacked; when the heat increases, and the periodical rains fall, the gastric symptoms become severe, but without diminution of the local affection of the head. As autumn advances, there is often an inflammatory affection of the intestines, frequently from improper treatment, terminating in dysentery. In the winter months, this disease is often accompanied by severe and evident inflammation of the lungs. In the summer and autumn, slighter affections of the lungs are occasionally observed, but the patient seldom complains of this, unless when asked.

"The symptoms which evince a more favourable termination, are the head-ach less severe in the first stage; the prostration of strength less remarkable; and the affection of the stomach more moderate; the pulse soft, and less frequent; and a gentle *uniform* moisture covering the whole body. The absence during the progress of the disease of the severe gastric symptoms, of the yellow suffusion, of ischuria, dyspnœa, singultus, and subsultus tendinum; the bowels moderately open, without pain on pressure.

"There are few severe cases when the disease is protracted beyond the third day, and in which the gastric symptoms are urgent, that the yellow suffusion does not make its appearance in; and the earlier it is observed, and the deeper hue it assumes, so in proportion is commonly the danger of the patient; not only as to his present recovery, but also as to the ultimate consequence of the fever; as in almost every instance it portends a protracted convalescence, and not unfrequently is followed by a diseased state of the liver, dropsical swellings, or irregular attacks of intermittent fever, probably depending ing on a morbid state of the brain, or other viscera. The foundation of phthisis pulmonalis is often laid by this disease, and the patient, though saved from the immediate, is destroyed by the remote effect of it."

*General Appearances on Dissection.*

" *External.*—The bodies of such as died during the inflammatory stage of the disease, were in general more slightly tinged with yellow. The yellow suffusion of those who died at a more advanced period, was found of a deeper hue. A dark livid appearance on the shoulders, and extending upwards to the occiput, was common to all. In several, livid blotches on different parts of the body; in a few, large black lines on the abdomen; the scrotum frequently quite livid. Black or reddish blotches on such parts as had suffered compression were common. In some petechiæ; abdomen tense; in a few, swelling and suppuration of the parotids.

" *Brain.*—Vessels generally distended; in many instances completely gorged with blood. The membranes highly inflamed, with often a blood-shot appearance. Depositions of coagulable lymph in different parts, particularly in the circumvolutions of the brain. Adhesions of the hemisphere were common. The ventricles often distended with fluid, sometimes limpid, at others yellow. Membranes of the brain frequently yellow. Substance of the brain of a firm consistence.

" *Thorax.*—The lungs often in a high state of inflammation; at other times effusion had taken place, and depositions of coagulable lymph on different parts. Adhesions to the pleura costalis were frequent. Pericardium inflamed, and preternatural collection of fluid within that membrane. Diaphragm highly inflamed, with occasionally depositions of coagulable lymph on its surface. Effusion to the extent of several ounces in the cavity of the thorax.

" *Abdomen.*—Liver generally enlarged, but not so commonly, showing great external marks of inflammation; frequently livid towards the lower edge of its concave side.

" Gall-bladder moderately full of inspissated bile.

" Stomach generally more or less inflamed and distended with air, containing a dark-coloured matter, adhering at times to its villous coat.

" Intestines bearing marks of inflammation through their whole course, distended with air, and containing a matter similar to that found in the stomach; frequent intussusceptio.

" Urinary bladder seldom distended, at times showing slight marks of inflammation."

The treatment proper to be adopted may be collected from the preceding observations. Bleeding, cold air, cold affusion, and the antiphlogistic regimen, seem to be indispensable.

Emetics, we think, are very properly objected to; and the author cautions the young practitioner not to trust solely to the use of the *cold or tepid affusion*, which are *most valuable auxiliaries*, but never of themselves to be depended on.

The

The use of mercury, except as a purgative, has never been found in the author's practice to be attended with benefit. *Many who were labouring under venereal disease, and whose systems were completely under the influence of mercury, were attacked with the same fever.* The pediluvium was found to increase the symptoms.

The remaining part of the work is devoted to the consideration of the fevers of Carthagena and Gibraltar, and the conclusions drawn from a great mass of evidence adduced on the subject, favour the opinion that these diseases are of the same nature with those of the Mediterranean, and consequently require the same treatment. The fatality of the Gibraltar fever is scarcely to be equalled in the annals of history. In *one epidemic only*, out of fourteen thousand inhabitants, five thousand nine hundred and forty-six died, being upwards of two-fifths of the whole population. Will it be credited that a fever, characterised by *violent pain in the head (confined chiefly to the eye-balls and forehead)*, back, and calves of the leg, flushed face, eyes shining, inflamed, and watery, like those of a person half drunk, could be suffered to run its course without the use of the lancet? Too true it is, and five thousand nine hundred and forty-six died. In justice to Dr. Burnett, we should observe these were not his patients.

The observations which follow are intended to refute the notion that this disease is of a contagious nature, an opinion previously asserted, if *not satisfactorily proved*, by Dr. Bancroft and others. The evidence brought in support of this opinion seems very conclusive, and we sincerely hope that this volume will find its way at least into the library of every army and navy surgeon.

---

*Commentaries on the Treatment of the Venereal Disease, particularly in its exasperated State; including a second Edition of a former Publication on that Subject, considerably augmented and improved. On the Use of Mercury, so as to ensure its successful Effect. With an Appendix, on Strictures of the Urethra, and on Morbid Retention of Urine.* By EDWARD GEOGHEGAN, Member of the Royal College of Surgeons in Ireland, Honorary Member of the Royal Medical Society, Edinburgh, and of the Physico-Chirurgical Society, Dublin. 8vo. pp. 219. Cumming and Co. Dublin, 1814.

(Continued from p. 417.)

HAVING pledged ourselves in our last Number to continue the analysis of this interesting volume, we resume the subject; ject; but as we find the remaining pages fall infinitely below the former in practical importance, we shall not detain our readers by any detail of the sentiments maintained in them. We find nothing to which we particularly object, but, on the other hand, little to commend on the score of novelty.

The author proceeds to give an account of what he considers the best mode of administering mercury so as to ensure its successful effect; after which the subjects of stricture and retention of urine are severally treated.

The practice recommended in the former is that of dilatation by *gum catheters*. It is admitted, we believe, almost universally, that nothing more is necessary for the cure of this disease in its simple form than the daily introduction of a bougie; and it is well known that the use of caustic is not in general had recourse to by the *best* and *most experienced* practitioners of this metropolis. The utility of dilatation cannot be denied, but we doubt whether a catheter can be made sufficiently small for some cases. In the worst instance of the disease we ever met with, the cure was commenced by a piece of whalebone cut to a much finer point than any bougie sold in the shops, which possessed the important advantage of firmness, unalterable by the warmth of the urethra. Something of this kind must necessarily be substituted for the plaster bougie, or gum catheter, when the obstruction is very great.

Where the stricture is seated at the curvature of the urethra, much benefit will be derived from the use of sounds made of different sizes, according to the degree of obstruction.

---

*Medico-Chirurgical Transactions, published by the Medical and Chirurgical Society of London. Volume the Fourth.* 8vo. pp. 490.—Longman and Co. 1813.

The appearance of this volume so soon after the last, at once proves the industry of the society, and the high estimation in which it is held by the profession. We shall notice the papers in the order of their insertion.

I. *Observations on the Venereal Disease in Portugal, as affecting the Constitutions of the British Soldiery and Natives.* By W. Ferguson, Esq. Inspector-General of Portuguese Military Hospitals.

This paper contains some interesting speculations on the complaint, and will be perused with interest, more, however, as a matter of historical curiosity, than for the practical improvement provement which it suggests, It is a curious fact observed by the author, that whilst the disease is more obstinate and intractable among our soldiers in Portugal, the natives suffer but little from its ravages, and for the most part require only topical treatment, without any adequate use of mercury. The conclusions which the author has arrived at, and which from his known talent and excellent opportunity for observation we have no doubt are correct, are as follow:

"1. That the disease, in its primary state, is curable in Portugal without mercury.

"2. That the anti-syphilitic woods, combined with sudorifics, are an adequate remedy for constitutional symptoms; the quantity of mercury being always insignificant, and often altogether omitted; or,

"3. That the virulence of the disease has become so much mitigated by reason of general and inadequately resisted diffusion, or other causes, that, after running a certain (commonly a mild) course, through the respective orders of parts, according to the known laws of its progress, it exhausts itself, and ceases spontaneously."

This paper affords an interesting addition to the numerous facts already recorded of that curious disease, which, from its varying character and continual recurrence, must still claim the attention of medical men, and upon which much may yet be written.

II. *Case of Paralysis of the Face, succeeded by certain Nervous Disorders.* By E. PERCIVAL, M.D. Physician to the House of Industry, Dublin.

A variety of the strange affections, generally called nervous, to which females are occasionally liable.

III. *An instance of Spasmodic Affection of the Tongue and Mouth, successfully treated.* By Mr. JOHN MITCHELL, Surgeon, of Kington, Herefordshire.

This case occurred in a female aged fifty; the spasmodic and muscular contraction was violent; appeared consequent on carious teeth, and was not cured till these were removed, and the gums freely scarified.

IV. *Two Examples of the beneficial Effects of Mercury in some severe Affections of the Brain.* By COLIN CHISHOLME, M.D. F.R.S.

"*Case* 1.—A female, aged 17, to whom I was called in August 1811, had for some time been affected with a pain in the region of the liver, accompanied with irregular symptoms of hysterics. The catamenia were regular in their periods and quantity. She was put on a course of mercury, and recovered from all her complaints. In

December following there was a relapse of these complaints; but the hysterical affections were more violent, for there were frequent paroxysms, in which, after strong convulsions, she fell into a state of mental derangement, consisting in delirious ravings and bursts of laughter. The natural functions were not interrupted; she had even a ravenous appetite. As the pain in the right hypochondrium continued, leading to a suspicion that an affection of the liver might have a connexion with these symptoms, the mercurial course was repeated, so as to produce a copious salivation. Under this all the symptoms disappeared. From an imprudent exposure to cold, before she had recovered her strength, all the same symptoms again returned. I had, on this occasion, an opportunity of verifying what Dr. Parry has alleged respecting the compression of the carotid arteries; for I found that this instantly, though temporarily, stopped the convulsions. On the first compression she fell into a state of syncope, and also on the second; but to this succeeded a state of general rigidity of the whole body and extremities, and a suspension of respiration, answering to the description of catalepsy. In ten minutes she started up in a state of mental distraction, which continued for ten minutes. She then relapsed into the cataleptic state; this alteration continued for an hour and ten minutes, and she then recovered her reason and recollection. Recourse was again had to the mercurial plan with complete ultimate success.

"*Case* 2.—A woman, aged 50, to whom I was called on the 1st June, 1812, had, about six weeks before, as I was informed, been suddenly seized with excessive head-ach and vertigo, dimness of sight, palpitation, sense of stricture across the *scrobiculus cordis*, great heat of skin, quick and strong pulse: there had also been a sense of suffocation and a fit of convulsion, which lasted for some hours, and was succeeded by *mania*. The whole paroxysm was completed in twelve hours. These fits returned daily. Evacuants, which I directed, seemed to relieve her, insomuch that she was free from fits till the 6th, when they recurred with much more violence than ever; for the most forcible means of coercion became necessary to restrain her under her tumultuous maniacal emotions. Encouraged by the favourable termination of the former case, I put her under a course of mercurial friction. In six days after commencing this, the paroxysms had entirely vanished, and she was restored to her reason. The friction having been continued so as to produce a ptyalism, she recovered her health, and remained well when I last saw her, which was the 27th of December."

### V. *Analysis of the Bones of the Spine in a Case of Mollities Ossium.* By John Bostock, M.D.

The result of this skilful experimenter's investigation is, that in mollities ossium 100 parts of the bone consist of

| | |
|---|---|
| "Jelly and oil . . . | 22 5 |
| Earthy matter . . | 20·25 |
| Cartilage . . . . . | 57·25 |
| | 100·00 |

"Human

"Human bones in their natural state, contain considerably more than half their weight of earthy matter; whereas the diseased bone in question contained in one part one-fifth only, and in another one-eighth of its weight."

VI. *Instance of the good Effects of Arsenic in Chorea.* By Mr. T. MARTIN, Surgeon, of Reigate.

In this case the arsenical solution succeeded in curing the complaint after purgatives and other remedies had failed.

VII. *An Example of Symptoms resembling Tic Douloureux, produced by a Wound in the Radial Nerve.* By ALEXANDER DENMARK, Esq. Surgeon to Haslar Hospital.

We insert this as a very interesting case, though very different from the disease called Tic Douloureux, which presents a regular series of symptoms peculiar to itself, and does not originate from external violence.

"Henry Croft, a healthy young man, belonging to the 52nd regiment, was wounded on the night of the 6th of April, 1812, at the storming of Badajos. A musquet ball entered the triceps extensor cubiti, about 1½ inch above the inner condyle of the os humeri, which, grazing the inside of that bone, passed obliquely downwards through the brachialis internus, and out anteriorly near the bend of the arm. The wound soon healed, and without manifesting any particular morbid symptom during the cure. On his admission into this hospital, I found him labouring under excessive pain, which the largest opiates could not assuage, with almost constant watching. The little sleep he had, if it could be called such, was disturbed by frightful dreams and starting. I always found him with the fore-arm bent, and in the supine posture, supported by the firm grasp of the other hand; the wrist also bent, being unable to move it in any other position by the voluntary exertion of its own muscles. He could suffer me to extend the hand, but with increased pain. It always, however, on the removal of the extending power, fell into its former bent situation. The act of pronation he could also suffer me to perform, but in like manner with increase of pain. A small tumor could be felt in the site of the wound on the anterior part of the arm, which he could not bear to be *touched* without evincing additional torture. He described the sensation of pain as beginning at the extremities of the thumb and all the fingers, except the little one, and extending up the arm to the part wounded. It was of a burning nature, he said, and so violent as to cause a continual perspiration from his face. He had an excoriation on the palm of the hand, from which exuded an ichorous discharge. The cause of this he ascribed to a shell rolling over it. His agonies, he observed, were insufferable, depriving him of sleep, and the enjoyment of his food, for which he had sometimes an appetite. He declared himself incapable of enduring it longer without some relief, and earnestly requested the removal of the arm. Before proceeding to any operation, I recommended him to try the effects of the warm and vapour baths, anodyne embrocations, &c. but from none of these he experienced any alleviation of his sufferings.

"The symptoms were sufficiently clear, I conceived, to lead to a correct prognosis. The part wounded, the nature of the pain, and its course from the fingers, with the exception of the little one, indicated the affection to be in the radial nerve. The increased pain attendant on the act of pronation further corroborated that supposition, from the pressure of the pronator teres upon the nerve in its passage through that muscle. The man said he had profuse bleeding after receiving the wound, yet the pulsation of the radial artery I found to be as strong as in the other arm. It was difficult to suppose the radial nerve wounded, and the humeral artery to escape: such, however, proved to be the case.

"I proposed to my patient the possibility of saving the limb, and relieving the pain, by cutting down upon the nerve, and removing a part of it, above the wound; which he willingly consented to, but observed, that he would rather have the arm amputated at once, than run the risk of a second operation. In a consultation which I held with my colleagues upon this case, when we considered the chance of failure, together with the injured state of the arm, and contracted elbow joint, we determined on the propriety of amputation. I immediately performed the operation, and with instantaneous relief to my patient. He was discharged cured in three weeks, having, in that time, rapidly recovered both his health and strength.

"On dissecting the arm, I traced the radial nerve through the wounded parts. It seemed to be blended with, and intimately attached to them, for the space of an inch. It had been wounded; and at the place of the injury was thickened to twice its natural diameter, and seemed as if contracted in its length. This contraction, I thought, partly accounted for the bent position of the arm, and the increased pain on attempting its extension; but on further examination, I was surprised to find, on dividing the fibres on the posterior part of the wounded nerve, that there was a small portion of the ball firmly imbedded in it, which had been driven off by grazing the bone. This description of injury more fully accounts for the exquisite pain felt by the patient. The os humeri was discoloured where it was grazed by the ball, and the humeral artery was uninjured! The nerve was evidently thickened, both above and below the wound. Would the division of the nerve, and cutting a piece of it out, have been attended with success?

"Mr. Bell relates a case of injured popliteal nerve, in a sailor, which he was about to operate upon, and which resembled very much the present, except that the injury was occasioned by contusion; in this, by wound."

VIII. *On the Nature and Analysis of Animal Fluids.* By JOHN BOSTOCK, M.D. Liverpool.

We have no hesitation in recommending this paper to those who are interested in such subjects of inquiry.

IX. *Ob-*

IX. *Observations on the comparative Prevalence, Mortality, and Treatment of different Diseases, illustrated by Abstracts of Cases which occurred to the Author at St Thomas's Hospital, and in his private Practice, embracing a period of Twenty Years.* By Sir GILBERT BLANE, Bart. M.D. F.R.S.

In this book-making age, it is highly commendable in the learned baronet, that he has condensed his information within the limits of 89 pages. Twenty years of extensive practice, public and private, would furnish most men with materials for as many volumes. His merit in observing brevity is the more admirable, because, independently of giving us the result of his own immediate experience, he commences with an historical account "of the most remarkable diseases which have arisen, and have since disappeared, in this country, in the course of time; of those which have arisen and not disappeared; and also of those which have prevailed with various degrees of frequency and fatality at different periods; concluding with an enumeration of those that have been more prevalent in our times than in former ages."

We can assure our readers that this part of the author's labours will afford them both amusement and instruction, but we cannot abridge what is already so much contracted. Sir Gilbert has drawn up a comparative statement of the diseases in the hospital, and those which he attended in private, which we shall insert in his own words.

"By comparing the number of the several diseases in the hospital list with those of the private list, it will be discovered which of them are most prevalent in the different ranks of society. Those which stand most prominent for this prevalence among the lower ranks are intermittent fevers, rheumatism, dropsy, and continued fever. One twentieth of the whole number on the hospital list were intermittent fevers, whereas* only one in one hundred and twenty-two belong to this head in the private list. Rheumatism constitutes one fifth part of the hospital list, but only one twenty-sixth of the private list. One case in nineteen of all the hospital list is a dropsy, but only one in fifty-nine of the private list. The difference here, as well as in the last mentioned, is clearly traceable to the habits of life. It is evidently imputable to the greater propensity of the lower orders to intoxication, particularly from the use of ardent spirits. Neither dropsy of the breast nor of the brain enter into this calculation. Of continued fevers there are about one in eight of the whole number on the

* In making this calculation, I have subtracted about five hundred from the total amount of private cases; for consumptions and small-pox are excluded from the hospitals, and a number of the catarrhs, children's complaints, and other cases, are such as would not have found admission as in-patients of the hospital.

the hospital list, and about one in eleven and a half in the private list. This may be easily accounted for from what has already been said of the usual origin of continued fevers.

"The diseases which stand most prominent for their prevalence among the upper classes of society are gout, disorders of the stomach, and liver complaints. With regard to gout, there is not a single case of it to be found on the hospital list, whereas there are in the private list a hundred and thirty, constituting about a twenty-sixth part of the whole. No disease affords so strong a proof of the power of habits of life over health.

"Disorders of the stomach constitute about a ninth part of the private list, but no more than a thirty-fifth part of the hospital cases. The reason of this is so obvious, and the fact itself so instructive, as to need no comment.

"The proportion of the diseases peculiar to the female sex in the hospital, is the same as in the private cases, from which it would appear, that the unfavourable influence of indolent habits, excessive delicacy and sensibility of mind and body in the upper ranks, compensates for the bad effects of hard labour and various privations in the lower orders, producing that equalization of human happiness and misery observable in other aspects of human life.

"Of liver complaints, about one in forty-three belong to the private list, and one in a hundred and thirty-three to the hospital list. This is partly owing to the greater proportion of the better sort, who come from tropical climates, and partly from jaundice and gall-stones, being complaints of more frequent occurrence in sedentary and indolent than in active and laborious life. It appears from the tables, that there is a considerably greater proportion of apoplexies and palsies among the hospital than among the private cases: this is what we should not at first sight expect, for it is matter of the most ordinary and superficial observation, that a much greater proportion of persons who live at their ease, especially of the male sex, are attacked with hemiplegia, particularly where it proves suddenly fatal, than of the laborious part of the community. One cause of the great proportion of them among the poor may be, that exposure to cold and wet in their necessary occupations is a frequent occasional cause of it among them, as I found by questioning them at their admission. Another cause of this great proportion of them being found in the hospital may be that these cases are so severe, so sudden and helpless, that they are all sent as speedily as possible to an hospital in the manner of accidents; and this is so true, that at St. Thomas's Hospital, an exception is made with regard to such cases, they are allowed to be considered as accidents, and are immediately admitted. Some cases of hemiplegia occur in full habits; some in spare and exhausted habits. The former being most incident to the luxurious and indolent, most frequently occur in private practice, and among the upper ranks of life. The latter occur more among the laborious classes, and among such of the rich as are addicted to exhausting pleasures.*

* See Lecture of Muscular Motion, page 29, read before the Royal Society, 1788, by Sir Gilbert Blane, M.D. F.R.S.

4 "With

"With regard to the two sexes, there appear to be certain diseases exclusive of those peculiar to each, which are more incident to the one than to the other. The proportion of the total females to the total males in the hospital tables, is not quite two-thirds; allowance being made for this, it will appear by inspection, that there is a considerable majority of males under the heads of intermittent fever, pulmonary complaints, bowel complaints, rheumatism, hemiplegia, other palsies, and dropsy. The only large head of disease in which there is a majority of females is cutaneous diseases. The cause of the great majority of intermittent fevers has been mentioned in the first article of the last volume of these Transactions. The reader will readily trace the causes of most of the other differences to the different constitutions and habits of life of the two sexes. With regard to the private cases, the number of each sex is not specified; but I find upon reviewing my notes, that they may be considered as equal. The diseases of which the great majority belong to the male sex, in the private list, are gout, pneumonia, asthma, rheumatism, palsy, especially that form of it called hemiplegia, the other species of palsy being nearly equal. There is a majority of male cases under dropsy, but much smaller than in the hospital list. I find the number of cutaneous cases equal in the two sexes, in my private notes, and am unable to assign any probable cause for the great proportion of such cases among the females at the hospital."

The author has demonstrated, from certain data, the advantage derived from the interference of art in the cure of diseases, and the whole paper may be regarded as favourable to his talents and industry. Tables are inserted containing the names of the diseases, and the final result, both in St. Thomas's Hospital and in the author's private practice, with illustrative observations.

In a supplement, Sir Gilbert Blane examines the question suggested by Dr. Watts's observations on the increased mortality of measles being influenced by vaccination. Although compelled to admit that of late years measles have proved more fatal than in former periods, we have pleasure in stating that, at least in London, the result of the present inquiry does not favour the opinion that vaccination is the cause of such an event; and, in a note, the author informs us that Dr. Stanger has ascertained, by an examination of the records of the Foundling Hospital, that out of a hundred and thirty-one patients who had undergone vaccination, and had afterwards had the measles, two only had died. It appears from the same records, that out of a hundred and thirty-one cases of measles in children who had previously had small-pox, eleven proved fatal.

X. On

X. *On the Effects of evacuating the Aqueous Humour in Inflammation of the Eyes, and in some Diseases of the Cornea.* By JAMES WARDROP, F.R.S. Ed.

This essay is important; the author suggests an improvement in the treatment of certain cases of ophthalmia, and affections of the cornea, of the highest moment, which in his hands has proved eminently successful. The memoir commences with the author's reasons for attempting the operation, and his explanation of the relief immediately afforded by it: he does not, however, insist that the discharge of the aqueous humour should be entirely relied upon in any case of ophthalmia, but regards it merely as a powerful auxiliary in some cases, in others as a sure and perhaps the only means of preventing the total destruction of the organ. He observes,

"When the object is to diminish suddenly the contents of the eye-ball, the evacuation of the aqueous humour must fulfil this intention in a more complete manner than we can conceive probable to be effected by any means we have of abstracting blood from its vessels; for, as the ophthalmic artery comes from the encephalon, little blood can be taken directly from any of its branches; and it would require a great quantity of blood to be drawn from the temples, or neighbouring arteries, to make any remarkable change in the quantity of the contents of the eye-ball; or at least a change equal to that which would be produced by the discharge of the aqueous humour. From the advantages also which have been universally found to arise from a sudden depletion of blood, in comparison with what can be derived from a slow detraction, considerable benefit might be expected from the practice now proposed; for as its effects must be immediate, a sudden change will be produced in the state of the organ, and a change favourable to the abatement of the inflammatory symptoms."

The mode of discharging the aqueous humour is thus described by Mr. Wardrop:

"The aqueous humour may be discharged by a very simple operation, nothing more being necessary than to make an opening through the cornea of a sufficient size to allow that fluid to escape, and in such a situation that any subsequent cicatrix may not impair vision.

"The opening may be made with any of the knives commonly used for extracting the cataract. It is sufficient that the point of the knife be introduced so that it makes a puncture into the anterior chamber; and this should be done near the junction of the cornea and sclerotic coat, at any part of the circumference. When the knife has penetrated the anterior chamber, it may then be withdrawn a little, and the blade turned on its axis, so that the aqueous humour will readily escape; and it is better not to remove the instrument altogether, till the fluid is observed to be discharged; for if the incision cision be not sufficiently large, and the knife taken away before the aqueous humour flows out, the elasticity of the cornea closes the wound, and either hinders the evacuation from being so sudden, and consequently so efficacious, or the closure of the wound entirely prevents its escape. The operation, therefore, which is necessary to discharge the aqueous humour, is merely the first step of the section of the cornea, made in extracting the cataract, or what has been called *the punctuation of the cornea.*

"The chief difficulty in performing the operation arises from the pain occasioned by the necessary pressure on the eye-ball, whilst keeping open the eye-lids; but until a sufficient portion of the cornea is brought into view, and the movements of the eye-ball completely under the management of the operator, the introduction of the knife should not be attempted.

"The upper eye-lid should be kept open either by the fingers of an assistant, or by Pellier's speculum. If the latter be employed, it will be found useful to have the silver wire covered with a piece of crape, which will prevent it slipping from any moisture of the skin, an accident very common, and very troublesome. The operator with the fore and middle fingers of one hand presses down the under eye-lid, and applies their points over the tarsi, in such a manner that they touch the eye-ball, and can apply any degree of pressure upon it which may be necessary. After the assistant raises the upper eye-lid, the patient should be directed to look downwards; and the assistant then employs a sufficient pressure, to keep the eye in that position.

"The operator may then make the puncture; but as the patient is very apt to start when he first finds the instrument coming in contact with his eye, I have found it useful merely to touch the cornea repeatedly with the back of the knife till all risk of starting is over; and as soon as the back of the extremity of the instrument rests on the part where the puncture is to be made, the knife can be raised very steadily on its point, and then the point thrust into the anterior chamber.

"Though I have described the method by which the puncture of the cornea may be made with a common extracting knife, yet it is evident that the aqueous humour may be discharged equally well by other instruments, such as a couching-needle; and of late I have been in the habit of generally employing the instrument of Mr. Cheselden. The more we are in the habit of using any particular instrument, the more dexterity and ease do we acquire in its use."

The operation has proved especially serviceable in cases of puriform ophthalmia, in severe cases of which the eye-ball sometimes spontaneously bursts, to the great and instant relief of the patient. Wherever this tendency is observed, the aqueous humor may be discharged with a certain expectation of success. Mr. Ware recommended the practice to the author, and Mr. Macgregor, surgeon of the York Asylum, has frequently practised it with great advantage. Out of

23 cases in which he operated, 21 were immediately relieved, and afterwards recovered their sight by the usual means.

Mr. Wardrop considers that the operation may be performed in any state and at any period of the disease, provided the accompanying symptoms are severe, and in children as well as adults; in short, in all cases where rupture of the cornea threatens.

He has enumerated several affections of the eye in which the operation may be performed, and illustrated them with cases, from which we would willingly give extracts, did not other papers in this volume claim our attention; and we hope also that Mr. Wardrop will shortly enable us to do his ingenuity more justice, when he publishes his treatise on diseases of the eye, in which we presume he will considerably enlarge upon the subject of his present memoir.

XI. *Case of Disease in the Brain, produced by external Violence.* By A. C. Hutchinson, M.D. Surgeon to the Royal Naval Hospital at Deal.

"Thomas Turnfield, a serjeant of marines, aged 36, was admitted into this hospital from his majesty's ship Dictator, on the 14th of April 1810, labouring under occasional attacks of stupor, as stated in his case transmitted by the surgeon of the ship. He was about five feet nine inches in height, muscular, of a sanguineous temperament, and without any marks of a scrophulous habit.

"Six years previous to this period, he was wounded on the left parietal bone by a cutlass, in boarding an enemy's vessel. The wound, by report, healed readily without any exfoliation, leaving a cicatrix two inches in length, parallel to, and a little to the left of, the sagittal suture.

"From the period of the infliction of the wound he had complained of a constant head-ach, which at the commencement was more or less acute, but in time became gradually obtuse.

"During the last seven or eight months he had been subject to fits of stupor, which came upon him at very irregular intervals; sometimes once or twice a week, at other times only once a fortnight, until about the beginning of March 1810, when the paroxysms became much more frequent, seldom lasting longer than an hour or hour and half, and he had always sufficient warning of their approach to lay himself down. During the intermissions he was perfectly sensible. His pulse, pupils, and countenance were natural. His appetite was good, and the kidneys continued to perform their functions naturally. His bowels, for the last two or three months, had been so torpid, as to require the powers of the strongest cathartics to move them.

"On the 15th April (the day after his admission), while I was in conversation with the patient, who was dressed, and had been walking about in the ward, he suddenly became silent, stepped a little aside, aside, and quietly laid himself down upon his bed. In less than three minutes his pupils were dilated to the utmost, and the iris of both eyes insensible to the stimulus of light. His pulse was slow and intermitting, not exceeding 52 strokes in the minute; his breathing laborious; and in half an hour he was foaming at the mouth.

" This paroxysm lasted a full hour and half; but in less than another hour he was walking about as before, free from every complaint, except slight languor, and his usual degree of head-ach.

" The patient died in one of those paroxysms, on the 19th of April, being the fifth day after his admission, and the fourth attack he had experienced while in the hospital.

" On the day of his admission, he had a brisk cathartic given him, and eighteen ounces of blood were extracted from the jugular veins, both which were opened. On the following day a seton was cut in the nape of the neck; his head was shaved, and directed to be frequently rubbed with a hard dry towel; and he was prescribed five grains of the Pilula Hydrargyri thrice a day.

" *Appearances on Dissection twelve hours after Death.*—The scalp had its natural appearance, and the cicatrix of the cutlass wound did not seem to adhere to the subjacent bone; but moved over it with the other parts, though in a somewhat less degree.

" On removing the scull-cap, the adhesions between the dura mater and bone were found stronger than usual; and the inner table of the scull, immediately under the cutlass wound, was free from any mark of disease.

" Upon detaching the dura mater from the brain, a portion of completely formed bone was found deposited upon its inner side, of the size of a finger nail, and tolerably thick. It was attached to the left side of the longitudinal sinus, and corresponded nearly with the direction of the wound on the scalp. The veins traversing the left hemisphere were rather more distended with blood than on the right; otherwise the surface of the brain possessed its natural appearance.

" On cutting into the substance of the brain, I found a tumour of a scrophulous nature, larger than a hen's egg, occupying a considerable portion of the middle lobe of the left hemisphere, and extending in depth to nearly on a line with the corpus callosum. The tumor was not detached from the adjoining cerebrum, but seemed merely as a condensed, or more properly indurated portion of brain. There was no appearance of pus, and the ventricles contained about an ounce of serum.

The plexus choroides and optic nerves were natural, the former containing very little blood. The other parts of the brain and cerebellum were free from any mark of disease.

" The thorax and abdomen, I lament to say, were not examined.

" That the morbid appearances just described originated in the wound on the scalp, we have, I think, considerable reason to believe—first, from their situation being immediately under the wounded part; and secondly, from the constant head-achs with which the patient was afflicted, having commenced at the period when the wound was received.

"Osseous deposits on the dura mater are not unfrequent; but it is worthy of remark, that this is the second instance I have met with, in which a deposition of bony matter on the side of one of the great sinuses has been accompanied by a tumour of the brain contiguous to it.*

"With regard to the mode of treatment to be pursued in such cases of organic affections of the brain as that now described, I confess myself not very competent to give an opinion. A course of mercury, at an early period of the complaint, as recommended by Sir Gilbert Blane, appears to me, for the reasons stated by that experienced physician, to be likely to be serviceable.†

"I cannot, however, here omit to urge the necessity of copious blood-letting, in the first instance, in all affections of the brain arising from external injury, concussion ‡ not excepted; and in such cases I do not know of any mode so good as that of puncturing the temporal artery. When it is found necessary to cut through the scalp, with the view to examine into the state of the bone underneath, I would also permit such vessels as may have been divided in the operation to bleed freely."

The author corroborates the propriety of this practice, in which we fully concur with him, by a case of fractured cranium, attended with considerable depression. The fracture was four inches in length, on the parietal bone, running from before backwards; and a depression of the inferior, or squamous part, about its middle, the whole thickness of the bone; but which gradually rose to its proper level as it approached the extremity of the fracture.

"Two small spiculæ of bone exfoliated, and in little more than a month the wound was cicatrised; but the violent attacks of head-ach and giddiness with which the patient was affected during the greater part of this period, called for so frequent a recourse to the lancet, that

---

* See the case of the gunner of the Fly, mentioned at p. 112 in the second volume of the Transactions of this Society.

† See a history of some cases of disease of the brain, with an account of the appearances upon examination after death, &c. by Sir Gilbert Blane, M.D. F.R.S. in the second volume of the Transactions of a Society for the Improvement of Medical and Chirurgical Knowledge. See also a valuable paper in the first volume of the Transactions of this Society, by Dr. Yelloly.

‡ In the extensive practice of some years at this public institution, I do not recollect a single instance in which I had reason to lament the practical conclusion. It is necessary to remark, however, that as all the cases admitted into the hospital come from ships of war lying in the Downs, some few hours generally elapse before the patient can be conveyed on shore, and thus an opportunity is afforded, during the interval, for vascular re-action to succeed the sedative effects of concussion.

on adding up, from his prescription ticket, the whole quantity of blood withdrawn at these different periods, within the first three weeks, I found it amount to nearly two hundred ounces, without taking into the calculation the quantity procured by the repeated application of leeches to the temples. He had also several blisters applied to the head; and latterly a seton was cut in the nape of the neck.

"In order to promote the absorption of the asperities of the depressed bone, which was beaten down upon the brain, and to round its edge, that the compression might be as slight as possible, the patient was put on a gentle course of mercury, which was continued nearly three months, during the greater part of which time his mouth was slightly affected; and on the 28th January 1813 he was discharged to his duty, free from every affection of the brain or other complaint."

In an appendix to the preceding paper, Dr. Hutchinson has related the particulars of a very curious case, which we here transcribe:

"Thomas Dawson, seaman, ætatis 28, of low stature, and of a full and healthy habit, while assisting to get up the top-gallant masts, on the 11th of March, 1813, the leading block gave way, and, his feet being entangled in the rope, he was tripped up, and his forehead struck with considerable violence on the deck. The part was contused and slightly discoloured, but the skin was scarcely abraded; he was a little stunned by the blow, but spoke sensibly soon after.

"On examination, no fracture nor fissure could be discovered; his pulse was regular, and his eyes natural; he had no nausea, but bled freely from the nose, and complained of a severe pain over the fore part of the head. He was prescribed a cathartic, and compresses wetted with Saturnine lotion were kept constantly applied to the contused part.

"In the afternoon he had slight nausea with vomiting; the head-ach was the same; and as the cathartic had not operated, it was directed to be repeated.

"*Second day.*—The symptoms continued nearly the same; the bowels not being sufficiently opened, the purgative was repeated. Towards evening, febrile symptoms began to show themselves; the pulse was increased in fullness and frequency; the skin was hot, face flushed, and eyes irritable. He had some stupor, with a disposition to sleep; the bowels were open. He was bled to twenty ounces, which entirely removed the febrile heat, and, in some degree, relieved the head-ach. Ever since the accident he had been unable to walk straight, and seemed to totter, not unlike a man intoxicated, the few times he tried that exercise.

"*Third day.*—He said the pain was chiefly confined to the posterior part of the head; the bowels were open; there was no febrile affection; and his eyes were less irritable. A blister was applied to the nape of the neck, the pediluvium used, and he took camphorated saline draughts every four hours.

"*Fourth day.*—There was no change, except that he rested rather better better throughout the last night than he had done since the accident: the camphorated saline draughts were directed to be continued.

" *Fifth day.*—He had considerable febrile heat, with much irritability and restlessness, and some thirst; his face was flushed; pulse frequent and rather hard; respiration hurried, and tongue white. He passed his urine unconsciously last night and this morning; pain now, he says, chiefly confined to the fore part of the head. He was bled again to 24 ounces, and, during this operation, the pulse kept its fulness. Immediately after the bleeding, he said his head was much relieved; the febrile heat, flushed face, and hurried respiration were certainly diminished, and he seemed more tranquil. The blood exhibited a dense buffy coat. Within a very few hours afterwards every symptom increased,—his pulse became small and frequent,—his pupils dilated,—low muttering delirium and restlessness succeeded, and at half-past twelve o'clock, on the sixth day (the 16th of March), he expired without the least convulsive struggle.

" It appears, by the concurring reports of his messmates, that he was always a healthy man, of a lively disposition, and not so much addicted to drunkenness as sailors generally are. It was stated that in the early part of his life he was in the capacity of a groom, when it appears he had been thrown from a horse, and had fallen upon his head with some degree of violence; but that during two years' service in the Christian he had never made any complaint. The immediate consequences of that accident could not be ascertained.

" *Dissection.*—On the 17th March the body was received at the hospital for interment, when I embraced the opportunity of examining the state and appearances of the brain.

" Upon inverting the scalp over the eyes and occiput, a slight degree of extravasation of blood was discovered a little above the superciliary ridge, where the blow had been received, but the bone was uninjured. The upper part of the skull being forced off, and the dura mater exposed to view, its vessels were found unusually distended. Between the os occipitis and posterior lobe of the right hemisphere, resting on the dura mater, about half an ounce of coagulated blood was found, which, by its remoteness from the injured part, was owing perhaps to the rupture of a small vessel, preternaturally distended with blood, a short period previous to dissolution. On removing the dura mater, the vessels of the pia mater were also unusually turgid.

" The right hemisphere, being divided on a level with the corpus callosum, and the ventricle exposed by another incision, six drachms of a colourless fluid escaped; and on examining this cavity, a portion of a small encysted tumour was found projecting into it at its anterior corner. The tumour was embedded in the corpus callosum, and a considerable part of it rested on the anterior crus of the fornix. It was of the size of a garden bean, felt smooth externally, and when pressed between the finger and thumb, communicated the sensation of its being filled with a cartilaginous substance.

" Its connexions were traced to the falx cerebri, and internal carotid artery, by the side of the sella turcica; and some very minute filaments filaments of vessels were also traced in the direction of the ophthalmic artery, where it enters the foramen. There was neither the appearance of pus nor inflammation in the vicinity of the tumour, and the surrounding brain exhibited no mark whatever of morbid affection.

"The left ventricle contained nearly two drachms of a colourless fluid, similar to that found in the right; but every other part of the brain and cerebellum appeared to be wholly free from disease.

"When the tumour was removed, and suspended by its numerous vessels, all of which entered together at one point, it exactly resembled a garden bean that had been planted a sufficient time for the radicle to have attained length to suspend it by,—the vessels entering where the radicle shoots.

"The cyst was opake and very strong, and contained a smaller cyst, the size of a barleycorn, imbedded in a semi-medullary and adipose substance, studded with minute portions of bony matter. The small cyst was filled with a piece of bone of an irregular round shape, composed of layers, which the preparation clearly demonstrates from a portion of the outer layer having been broken off by the instrument in laying open the cyst.

"That the tumour had existed a considerable period antecedent to the accident which was the immediate cause of the man's death, there is little doubt; and that the preternatural appearances observed on dissection, independently of the tumour, were sufficient to account for his dissolution, appears to me equally certain.

"This case, therefore, seems to evince, in addition to the evidence already existing upon this subject, that a morbid structure of the brain may exist for a long time without much inconvenience to the patient, or the slightest interruption to the various animal functions; and, with other cases on record,* is, in my estimation, sufficient to authorise the conclusion, that a variety of chronic, and other affections of the head, are referable to blows or falls received in early life; when, probably, the accident that originally occasioned them had wholly escaped recollection. If I mistake not, a similar observation was made by a gentleman present at the reading of my former paper on this subject to the Society.

"How long Dawson might have lived with the tumour on his brain, had the accident of which he died not occurred, is matter of mere speculation."

(To be continued.)

### MEDICAL AND PHILOSOPHICAL INTELLIGENCE.

AT a general meeting of Apothecaries, &c. held at the Crown and Anchor Tavern, Strand, on Thursday, May 12th, 1814, Mr. Burrows in the Chair, the following Report was brought up

* "See Dr. Lettsom's case in the 3d vol. of the Memoirs of the Med. Soc. of London, before quoted; Sir Gilbert Blane's, in the 2d vol. of the Transactions of a Society for the Improvement of Medical and Chirurgical Knowledge; and Serjeant Turnfield's case."

